# Application of mixture design methodology for development of high antioxidant fruity functional beverage

**DOI:** 10.1002/fsn3.2834

**Published:** 2022-04-21

**Authors:** Samar Sahraee, Babak Ghanbarzadeh, Pasquale M. Falcone

**Affiliations:** ^1^ Department of Food Science and Technology Faculty of Agriculture University of Tabriz Tabriz Iran; ^2^ Department of Food Engineering Faculty of Engineering Near East University Mersin Turkey; ^3^ Department of Agricultural, Food and Environmental Sciences Marche Polytechnic University Ancona Italy

**Keywords:** essential oil, extract, functional beverage, mixture design, optimization

## Abstract

Three red color fruit juice (pomegranate (PJ), barberry (BJ), and grape juice (GJ)) and three plant extracts (cardamom essential oil (CE), ginger extract (GE), and hibiscus solution (HS)) were used for the development of different functional beverages. Organoleptic analysis was done to detect the most acceptable fruit juice blend. The physicochemical properties of the samples including total phenols, 1,1‐diphenyl‐2‐picrylhydrazyl (DPPH) inhibition percent, anthocyanin, flavonoid, and vitamin C content of optimum fruit juice blend (60% PJ/20% BJ/20% GJ) were 121.57 µg gallic acid equivalent (GAE)/ml, 80.28%, 4.03 mg/L, 64.87 mg/100 ml, and 51.10 mg/100 ml, respectively. To determine the optimum level of extracts and essential oil (GE, CE, and HS) in fruit juice blends, the mixture design method was used and 14 runs (formulations) were obtained. In all formulations, samples containing HS had the highest content of antioxidant and active components and the statistical analysis indicated that the sample containing 0.5 CE/0.5 GE/1 HS (ml/100 ml) had the optimum content of antioxidant components. Thus, the results of this study introduce a functional drink possessing high polyphenols, antioxidants, anthocyanin, and vitamin C content.

## Graphical abstract

The mixture design methodology was used for the development of different functional beverages. Three red color fruit juice (pomegranate (PJ), barberry (BJ), and grape juice (GJ)) and three plant extracts (cardamom essential oil (CE), ginger extract (GE), and hibiscus solution (HS)) were used. Organoleptic analysis was done to detect the most acceptable fruit juice blend. The physicochemical properties of the samples including total phenols, 1,1‐diphenyl‐2‐picrylhydrazyl (DPPH) inhibition percent, anthocyanin, flavonoid, and vitamin C content of optimum fruit juice blend were measured.

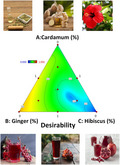

## 1 INTRODUCTION

Functional food is a relatively new term used to describe food products that have been enriched with bioactive components with a specific disease‐preventive and/or health‐promoting effect in addition to their fundamental nutritive value (Gunathilake et al., 2013). Blending fruits and vegetables juice is considered as one of the best methods to develop a functional beverage with improved nutritional quality.

Reactive oxygen species are formed naturally within the biological systems and can potentially create oxidative damage via interaction with biomolecules. The free radicals are also produced by the enzymatic reactions and by external sources, such as pollution, cigarette smoke, and sunlight (Dey & Sireswar, 2020; Han et al., 2007). When excessive free radicals are formed, cellular injury can occur, leading to a broad range of degenerative alterations, such as tissue degradation, cardiogenesis, aging, diabetes, neurodegenerative diseases, and other oxidative stress‐related diseases.

To decrease oxidative stress in the body, adequate antioxidants should be included in the diet (Ramadan‐Hassanien, 2008). Fruits and vegetables are important sources of antioxidant components and scientists believe that consumption of them routinely in the diet reduces the incidence of degenerative diseases (Dey & Sireswar, 2020; Dogan et al., 2020).

Pomegranate, barberry, and red grape are popular red color fruits and all of them are rich in polyphenols (especially anthocyanin) and can be applied for the development of antioxidant‐enriched functional fruit juices and drinks (Berenji & Akhavan, 2018; Derakhshan et al., 2018; Jaddi et al., 2020). Several studies have investigated the health‐promoting properties of pomegranate, barberry, and grape (Dimitrijevic et al., 2020; Mphahlele et al., 2016; Xu et al., 2010). Fahmy et al. (2020) reported that pomegranate juice is rich in dietary polyphenols like tannins, anthocyanins, and flavonoids which are effective in obesity and diabetes management. Also, grapes include phenolic compounds, anthocyanins, flavanols, stilbenes, catechins, and proanthocyanins (Nadeem et al., 2018). Nadeem et al. (2018) developed a grape–carrot juice blend because of the functional properties of these juices such as antioxidant, anticancer, and antianemic properties of carrot juice and reducing atherosclerosis threat of heart diseases of grape juice. In the case of barberry juice, its predominant organic acid is malic acid and it is rich in polyphenols like gallic acid, catechin, chlorogenic acid, and quercetin (Gundogdu, 2013). On the other hand, in recent years, the addition of herbal extracts and essential oils such as ginger extract (GE), cardamom essential oil (CE), and hibiscus solution (HS) to functional foods has been considered. These materials are of interest due to their high antioxidant, anticancer, and antimutagenic properties. Imran et al. (2021) developed a functional drink containing mint and ginger extract because of their high phenolic compounds and antioxidant activity. Studies showed that *Hibiscus sabdariffa* L. (HS) calyx extracts are effective in treating hypertension, liver dysfunctions, and diabetes which is due to their high phytochemical, anthocyanins, phenolic compounds, and organic acids (Salem et al., 2021). Ogundele et al. (2016) studied the effect of blending pineapple, orange juice, carrot, and *Hibiscus sabdariffa* extracts on antioxidant properties of beverages. In all blends, juices containing HS showed high antioxidant activity. Ogori et al. (2021) investigated the functional and quality characterization of ginger, pineapple, and turmeric juice blends. They concluded that increasing the turmeric juice proportion increased the Mg, Ca, and vitamin C content of blends. Also, turmeric and ginger extracts are very effective on the antimicrobial properties of the juices.

The mixture design is a practical design for optimization and choosing the best formulation of ingredients in a combination (Ogundele et al., 2016). This research work aimed to develop a new antioxidant functional beverage by blending red color fruit juices (pomegranate, grape, and barberry juices) with three plant extracts and essential oils (hibiscus extract, ginger extract, and cardamom essential oil) based on a mixture design method.

## 2 MATERIALS AND METHODS

### 2.1 Chemicals

Pomegranate, barberry, and grape concentrates were purchased from Takdaneh Co., Tabriz, Iran. Hibiscus powder, cardamom essential oil (CE), and ginger extract (GE) were purchased from Darou Gostar Barij Essence Co., Kashan, Iran. All chemicals applied in this study were of analytical grade and provided from Sigma‐Aldrich, Oakville, ON, Canada.

### 2.2 Preparation of blends of fruit juices

Seven fruit juice formulations with different proportions of grape, pomegranate, and barberry juices were blended (Table 1) and pasteurized at 92 ± 2°C for 3 min (the Brix of all blended juices was 12°). Juices were hot filled in the bottles, cooled, and stored at 4°C.

**TABLE 1:** Different blends of fruit juices used for initial sensory evaluation

Fruit juice	1	2	3	4	5	6	7
Pomegranate	33.33%	50%	25%	25%	60%	20%	20%
Grape	33.33%	25%	50%	25%	20%	60%	20%
Barberry	33.33%	25%	25%	50%	20%	20%	60%

### 2.3 Initial sensory evaluation

To determine the best proportions of fruit juices in the mixture and then, the maximum acceptable level of GE and CE, which could be added to the formulations, two steps of sensory evaluation were performed.

In the first stage, 30 ml of each seven juice blends (Table 1) was served in transparent plastic cups. Sensory evaluation of the beverages was done through a 9‐point hedonic scale where 1 equals extremely dislike and 9 equals extremely like (Gunathilake et al., 2013). The panelists consisted of 15 members (semi‐trained men and women students aged 23 to 30 years) who evaluated the desirability of taste, sourness, sweetness, stringiness, and overall acceptability of different juice blends.

In the second stage, to determine the maximum level of extracts and essence which can be added to the juice from the consumer point of view, 1 ml of three concentrations of CE (0.05, 0.01, 0.005 v/v) or GE (0.1, 0.01, 0.001 v/v) was added to 99 ml of the chosen juice blend (the amount of hibiscus did not lead to undesirable taste and odor up to high levels in the juice, so it was not sensory evaluated). Again, as described above, a 9‐point hedonic test was conducted to estimate the maximum amount of plant extracts acceptable as consumer sensory perception. Taste, odor, and overall acceptability were evaluated for the samples. Statistical analysis of the data was carried out through analysis of variance (ANOVA) and significant differences were estimated by the Duncan test.

### 2.4 Experimental design for the formulation of functional beverages

To develop functional fruit juices, the D‐optimal mixture design with no blocking was applied. The number of independent factors (proportion of CE, GE, and HS) determined the number of points in the design. Total phenols, 1,1‐diphenyl‐2‐picrylhydrazyl (DPPH) inhibition activity, flavonoid, anthocyanin, and vitamin C content were the responses influenced by the changes in the proportion of independent variables. The statistical software package Design‐Expert 11.0.3.0, Stat‐Ease, Inc., Minneapolis, MN, USA, was applied for determining the experimental design and analyzing the data. Table 2 shows the coded values and actual values of GE, CE, and HS to be added to the juices. The center points of these designs were chosen to add ingredients to the juices to yield satisfactory experimental results. The functional juices were obtained by the addition of different proportions of GE, CE, and HS (with predetermined concentrations) to the fruit juice blends and investigated for their physicochemical properties. The design resulted in 14 experimental runs with 4 replicates (Table 3). The data of responses were analyzed applying statistical software for selecting the best fit design and optimized formulation.

**TABLE 2:** Experimental ranges of independent variables applied in D‐optimal mixture design for the formulation of functional fruit juice blend

Variables	Range of levels			
	Low actual (ml/ 100 ml of juice)	Low coded	High actual (ml/ 100 ml of juice)	High coded
Ginger extract (0.001 v/v)	0	+0	1	+1
Cardamom essential oil (0.005 v/v)	0	+0	1	+1
Hibiscus solution (0.002 w/v)	0	+0	1	+1
Total	2			

**TABLE 3:** Optimal mixture design matrix for the addition of plant extracts to the fruit juice

Formulation	Ginger solution (0.001 v/v)	Cardamom solution (0.005 v/v)	Hibiscus solution (0.002 w/v)
S1	0.66	0.66	0.66
S2	1.00	0	1.00
S3	1.00	1.00	0
S4	0	1.00	1.00
S5	0.83	0.83	0.34
S6^a^	0	1.00	1.00
S7	1.00	0.50	0.50
S8^a^	0.66	0.66	0.66
S9	0.83	0.34	0.83
S10	0.50	0.50	1.00
S11^a^	1.00	1.00	0
S12	0.34	0.83	0.83
S13^a^	1.00	0	1.00
S14	0.50	1.00	0.50

Note

All the values are in milliliters (ml) to be added to 100 ml fruit juice.

^a^ Replicates.

### 2.5 Physicochemical properties

#### 2.5.1 pH, total soluble solids, and total acidity

The pH of beverage samples was analyzed using a pH meter (Fan Azma Gostar, Iran). A handheld refractometer was used for estimating the total soluble solids (g/100 g) of juices. The total acidity of the samples was measured using the titration method (Cassani et al., 2016). Five milliliters of samples was transferred to a 250 ml beaker and 50 ml of boiled distilled water was mixed with the sample. This solution was titrated by NaOH (0.1 N) up to the pH of 8.2 ± 0.1. Total acidity was calculated according to citric acid (g/100 g) using Equation (1):

(1) $$\text{Acidity}=\frac{V\times 0.1\times 0.064\times 10}{S}$$

where *V* is the volume of NaOH and *S* is the volume of the juice sample.

#### 2.5.2 Total phenolic content (TP)

Total phenolic content (TP) was determined according to the method of Kowalski and Gonzales de Mejia (2021) using the Folin–Ciocalteu reagent (FCR). The calibration curve was prepared using gallic acid (0–100 µg/ml). One milliliter of diluted samples (1/10) or gallic acid was mixed with 5 ml of FCR (10%). Then 4 ml of saturated sodium carbonate was added to the solution and kept for 2 h at room temperature. The absorbance of samples was determined at 765 nm using a ultraviolet–visible (UV–Vis) spectrophotometer (UV‐1700, Shimadzu, Japan) and the total phenol (TP) was estimated as mg of gallic acid equivalent (GAE) per 100 ml of sample.

#### 2.5.3 Total flavonoid content

Total flavonoid content was investigated according to (Orellana‐Palma et al., 2021) with some modifications. As much as 0.2 ml of diluted samples (1/10) was added to 1.28 ml of distilled water and 60 µl of NaNO_2_ (50 g/L) and kept for 5 min at ambient temperature. Sixty microliters of AlCl_3_ (100 g/L) was mixed with the solution and after 6 min, 0.4 ml of NaOH (40 g/L) was added to the solution. After stirring the mixture, the absorbance was determined at 510 nm applying a UV–Vis spectrophotometer. Total flavonoid content was stated as mg of quercetin equivalent/100 ml of the sample according to the standard curve prepared in the range of 0.05–1.2 g/L.

#### 2.5.4 DPPH radical scavenging activity

The antioxidant property of samples was studied through DPPH radical scavenging activity according to the method of Viacava and Roura (2015) with some modifications. As much as 0.1 ml of diluted samples (1/10) was mixed with 3.9 ml of DPPH solution (100 µM). After shaking the solution, it was kept in the dark place at ambient temperature for 30 min. Ethanol (96%) was used instead of the sample in the above procedure to determine DPPH initial absorbance. Antioxidant activity of the samples was reported as the inhibition percent of the DPPH radical according to Equation (2).

(2) $$\text{Inhibition(\% )}=\left[\left(A_{c}‐A_{s}\right)/A_{c}\right]\times 100$$

Where *A_c_* is the absorbance of the control (methanol and DPPH solution) and *A_s_* is the absorbance of the sample solution (sample and DPPH solution).

#### 2.5.5 Anthocyanin content of samples

Polymeric anthocyanins are resistant to pH change and color degradation and therefore do not participate in adsorption by spectrophotometry. In contrast, the monomeric pigments of anthocyanins are unstable against pH changes. Oxonium forms of anthocyanins become colorful at pH = 1 and hemiketals become colorless at pH = 4.5. Therefore, sample absorbance was measured at pH = 1 to determine the total anthocyanin content and at pH = 4.5 for quantification of polymeric anthocyanins. Finally, total monomeric anthocyanins are determined from differences in absorbance at 520 and 700 nm. The results were expressed as cyanidin 3‐glucoside equivalents calculating with Equations (3) and (4):

(3) $$\mathrm{Anthocyanin}\left(\frac{\mathrm{mg}}{L}\right)=\;\frac{A\;\times \mathrm{Mw}\;\times \mathrm{Df}\;\times 1000}{\varepsilon \;\times L}$$

(4) $$A=\left(A_{520}‐A_{700}\right){\text{pH}}_{1}‐\left(A_{520}‐A_{700}\right){\text{pH}}_{4.5}$$

Where Df is the dilution factor, MW is cyanidin 3‐glucoside molecular weight (449.2 g/mol), A is the absorbance of the sample, *ɛ* is the molar extinction coefficient (26,900 L/cm mol), and L is the cell length (1 cm; Monteiro et al., 2016).

#### 2.5.6 Vitamin C content

Vitamin C content of fruit juices was measured according to the method described by Tareen et al. (2015). In this method, 5 ml of juices was transferred to a 125 ml Erlenmeyer flask and diluted with 45 ml of distilled water. Then, 10 drops of indicator (1% starch solution) were added to the juice. This solution was titrated by iodine solution (0.005 mol/L) until the color of the solution changed to blue‐black. The iodine solution was prepared as follows: 2 g of potassium iodide (KI) was dissolved in 100 ml of distilled water. Then, 1.3 g of iodine was added to the solution. Finally, the solution was diluted with distilled water until it reached the 1000 ml volume (Tareen et al., 2015). Vitamin c concentration is calculated according to Equation (5):

(5) $$\text{Vitamin C}\left(\text{mg}/100\;\text{ml}\right)=10\times 17.6\times V$$

Where *V* is the volume of iodine solution used in the titration.

### 2.6 Statistical analysis

All experiments were repeated four times and the data were subjected to statistical analysis using Design‐Expert version 11.0.3.0 by Stat‐Ease Inc., Minneapolis, MN, USA. A predictive model that can accurately describe the response was selected based on the quality of fit estimated by ANOVA.

## 3 RESULTS AND DISCUSSION

### 3.1 Physicochemical properties of different blends of fruit juices

The results of physicochemical analysis of different fruit juice blends are shown in Table 4. The total soluble solids of all of the seven juice blends were approximately 12° Brix with no significant difference (*p* < .05). Blend 4 (25% PJ, 25% GJ, and 50 BJ) and blend 7 (20% PJ, 20% GJ, and 60% BJ) had the lowest pH value and highest acidity. In terms of active compounds’ content, blends 7 and 5 (60% PJ, 20% GJ, and 20% BJ) had higher phenolic content and DPPH radical scavenging capacity (*p* < .05). On the other hand, blends 3 and 6, which had higher grape juice portions, showed lower phenolic content and antioxidant property than other blends. Significantly higher vitamin C content belonged to blends 2 and 5 having higher portions of PJ, while blend 7 had lower vitamin C than other blends. Blends 4, 5, and 7 had significantly higher total flavonoid and anthocyanin content than other blends and had no significant difference between each other. Blend 6 showed the lowest amount of flavonoid and anthocyanin content.

**TABLE 4:** Physicochemical properties of different fruit juice blends

Properties	Blend 1	Blend 2	Blend 3	Blend 4	Blend 5	Blend 6	Blend 7
Acidity (%)^1^	0.90 ± 0.01^a^	0.90 ± 0.02^a^	0.88 ± 0.02^a^	0.93 ± 0.01^b^	0.91 ± 0.01^a^	0.90 ± 0.01^a^	0.95 ± 0.02^b^
pH	3.08 ± 0.02^b^	3.10 ± 0.01^b^	3.19 ± 0.03^c^	3.02 ± 0.02^a^	3.14 ± 0.03c	3.17 ± 0.02^c^	3.00 ± 0.01^a^
Total phenols (µg/ml)^2^	111.78 ± 0.96^b^	114.29 ± 1.04^c^	82.12 ± 2.11^a^	108.95 ± 2.48^b^	121.57 ± 3.71^d^	79.90 ± 1.23^a^	118.24 ± 1.47^d^
DPPH scavenging activity (%)^3^	78.12 ± 0.37^c^	77.43 ± 0.78^c^	32.46 ± 0.59^b^	77.96 ± 0.81^c^	80.28 ± 0.29^d^	25.14 ± 1.07^a^	81.33 ± 0.85^d^
Vitamin C (mg/100 ml)	45.55 ± 0.99^d^	49.58 ± 2.01^e^	39.64 ± 2.48^b^	40.33 ± 0.88^c^	51.10 ± 0.95^e^	42.49 ± 1.73^c^	35.11 ± 1.09^a^
Flavonoid content (mg/100 ml)^4^	62.04 ± 0.83^b^	61.46 ± 0.96^b^	60. 98 ± 1.49^b^	64.07 ± 2.54^c^	64.87 ± 1.99^c^	58.22 ± 1.50^a^	66.23 ± 2.11^c^
Anthocyanin content (mg/L)^5^	3.75 ± 0.04^c^	3.80 ± 0.07^c^	3.37 ± 0.11^b^	4.00 ± 0.05^d^	4.03 ± 0.07^d^	3.12 ± 0.08^a^	3.99 ± 0.10^d^

Note

All values are means ± *SD*, *n* = 3.

Different superscript in each row shows the significant difference between values (*p* < .05).

^1^ mg of citric acid equivalents/ 100 ml.

^2^ µg of gallic acid equivalents/L.

^3^ Inhibition percent.

^4^ mg of quercetin equivalents/100 ml.

^5^ mg of cyanidin 3‐glucoside equivalents/L.

### 3.2 Evaluation of sensory properties of fruit juice blends

In this section, panelists scored fruit juice blends for their acceptability of sourness, sweetness, stringiness, and overall acceptability (Table 5). Blend 7 gains the lowest score for its sourness, sweetness, and overall acceptability. According to the results, blends 4 and 7 were too sour to be acceptable. Probably, it is because of the high proportion of barberry juice in the blend. The highest sweetness was recorded in blends 3 and 6, which had more grape juice. In terms of overall acceptability, blends 2 and 5 were similar and gained the highest scores in comparison with other blends. So, according to physicochemical properties and sensory evaluations, blend 5 with 60% pomegranate juice, 20% grape juice, and 20% barberry juice was chosen as the final blend to be added CE, GE, and HS.

**TABLE 5:** Sensory evaluation of different fruit juice blends

Fruit juices	Acceptability scores			
	Sourness	Sweetness	Stringiness	Overall acceptability
Blend 1	4.20 ± 0.31^ab^	4.50 ± 1.8^a^	7.80 ± 0.94^b^	5.90 ± 1.01^b^
Blend 2	8.50 ± 1.01^d^	9.00 ± 0.51^c^	8.50 ± 1.23^b^	9.30 ± 0.45^c^
Blend 3	6.50 ± 0.93^c^	8.00 ± 0.33^b^	7.50 ± 2.11^ab^	6.70 ± 1.84^b^
Blend 4	4.50 ± 0.23^b^	2.50 ± 2.39^a^	6.20 ± 0.83^a^	5.30 ± 1.59^ab^
Blend 5	8.90 ± 0.72^d^	9.10 ± 0.69^c^	8.20 ± 0.99^b^	9.40 ± 0.55^c^
Blend 6	6.50 ± 0.79^c^	7.20 ± 1.04^b^	6.40 ± 1.55^a^	7.80 ± 1.08^b^
Blend 7	3.80 ± 0.20^a^	2.50 ± 2.77^a^	5.90 ± 0.91^a^	4.20 ± 0.50^a^

Note

All values are means ± *SD*, *n* = 3.

Different superscript in each row shows significant difference between values (*p* < .05).

Higher values of sourness, sweetness, stringiness, and overall acceptability indicate more liking of the characteristic.

### 3.3 Determination of the maximum acceptable level of extracts

Sensory evaluation of fruit juice blends containing different concentrations of GE (0.1, 0.01, 0.001 v/v) or CE (0.05, 0.01, 0.005 v/v) was performed to investigate the maximum acceptable amount of extracts that could be added to the fruit juices (Table 6). According to the results, blends containing higher concentrations than 0.001 (v/v) of GE gain lower scores of taste and odor, significantly. In this regard, the overall acceptability of blend containing 0.001 (v/v) GE was higher than the other ones. Also, according to the panelists’ scores, the maximum acceptable concentration of cardamom essential oil (CE) in terms of taste and odor in the fruit juice blends was 0.005 (v/v).

**TABLE 6:** Sensory evaluation of fruit juice blends with different levels of ginger extract or cardamom essence

Levels of additives (v/v)	Sensory scores		
	Taste	Odor	Overall acceptability
Ginger extract			
0.001	7.5 ± 1.1^b^	8.0 ± 0.8^b^	7.7 ± 0.5^b^
0.01	5.2 ± 0.9^a^	5.7 ± 1.3^a^	5.0 ± 1.4^a^
0.1	4.3 ± 1.0^a^	3.9 ± 0.6^a^	3.7 ± 0.9^a^
Cardamom essence			
0.005	8.5 ± 0.4^b^	7.2 ± 0.5^b^	7.5 ± 0.9^b^
0.01	5.2 ± 1.6^a^	4.0 ± 1.0^a^	4.7 ± 1.8^a^
0.05	4.9 ± 0.7^a^	3.7 ± 0.6^a^	3.8 ± 2.1^a^

Note

Values with different superscripts in each column are significantly different.

Values are presented as mean ± *SD*.

### 3.4 Optimization of antioxidant functional beverage

In the first stage of this research work, selecting optimum fruit juice blend based on physicochemical and sensory properties and determining the maximum level of ginger extract and cardamom essential oil were carried out. In the second stage, different formulations of functional beverages were prepared according to the mixture design method with the blending of different levels of three extracts and the optimum fruit juice blend (Table 2).

#### 3.4.1 TP and DPPH radical scavenging activity of beverage formulations

Analysis of variance (ANOVA) was used with the purpose to find out how three plant extract levels affected the total phenol (TP) and DPPH values. It was observed that the model had a very high *F*‐value (*F* = 91.77) for TP response, implying that the quadratic regression equation (Equation (6)) was extremely significant and incorporation of extracts into fruit blends had a significant effect on the total phenol content at *p* ≤ .05 (insignificant lack of fit = 0.42).

(6) $$\text{TP}=169.67A+157.56B+133.77C‐37.57\text{AC}‐689.75A^{2}\text{BC}+687.43{\text{ABC}}^{2}$$

Where A is CE, B is GE, and C is HS.

The high determination coefficient (*R* ^2^) and adjusted *R* ^2^ values (0.9932 and 0.9824, respectively) indicated that the three independent parameters could well describe the majority of changes in the dependent variable (TP). According to the table of ANOVA for the special quartic model, the interaction effect of AB, BC, and AB^2^C was insignificant in Equation (6), and so these factors were deleted.

**FIGURE 1:**
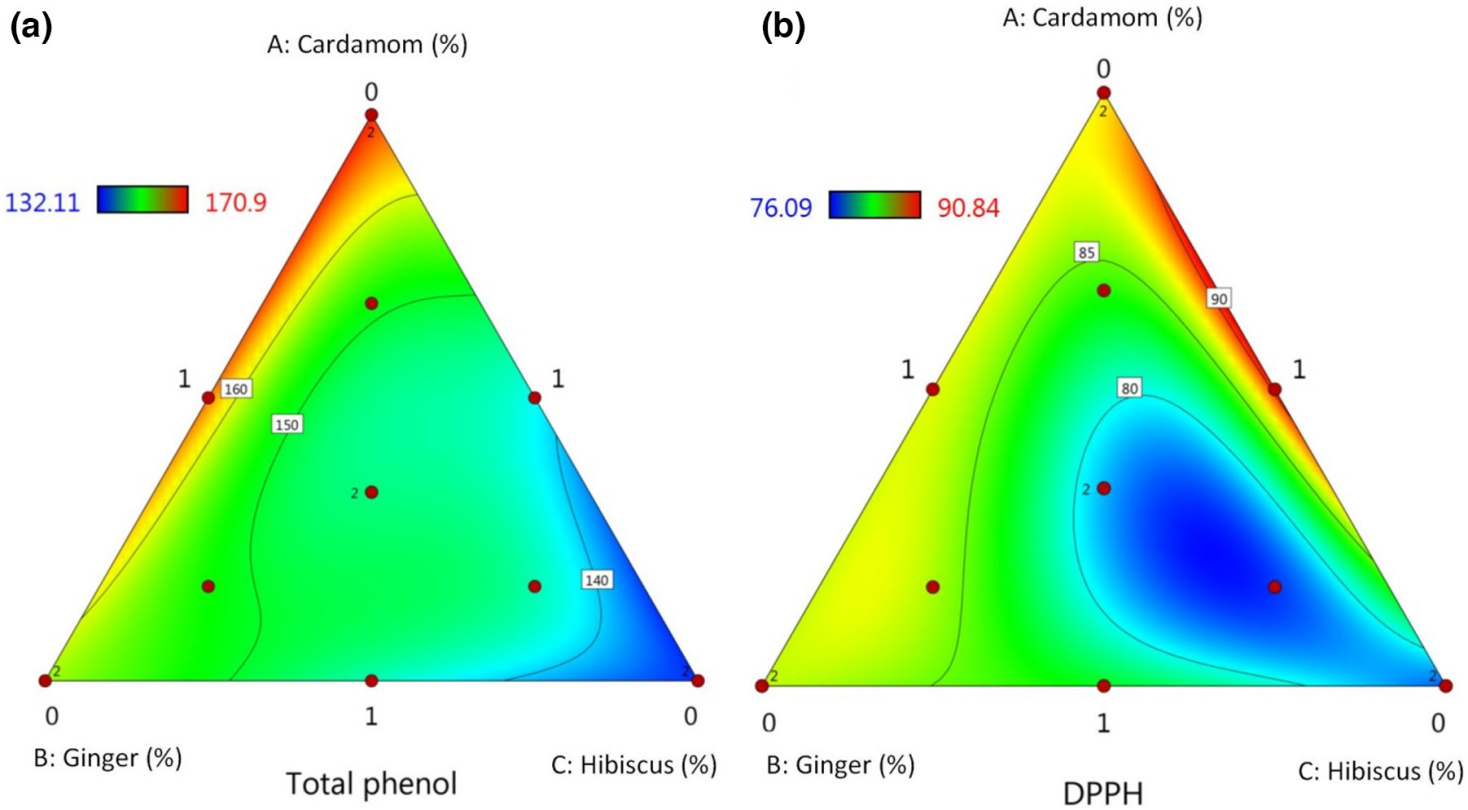
Plots showing the effect of ginger extract (GE), cardamom essential oil (CE), and hibiscus solution (HS) on total phenol content (TP) (a) and 1, 1‐diphenyl‐2picrylhydrazyl (DPPH°) inhibition percent (b) of fruit juice blends

The contour diagram (Figure 1a) showed that TP content was mainly dependent on the ginger and hibiscus concentrations in fruit juices (S2, S9, and S13 samples). The response plots for TP indicate that a higher amount of TP is detected in juices with a higher concentration of ginger and hibiscus (maximum TP was 170.9 µg/ml for 1 ml GE/1 ml HS/0 CE juice blend and minimum TP was 132.11 µg/ml for 1 ml GE/1 ml CE/0 hibiscus). There were high amounts of TP in juices even at low concentrations of cardamom essential oil (Figure 1a). Essential oils are mainly composed of terpenic compounds and usually have lower polyphenols than plant extracts. Terpinyl acetate and 1,8‐cineol are two major compounds containing about 70% of cardamom essential oil (Amma et al., 2010), which are monoterpenoid compounds. While gingerols, shogaols, and paradols are primary polyphenols of ginger extract (Mao et al., 2019). Polyphenols include different secondary metabolites with aromatic rings that have one or more hydroxyl moieties and are foremost responsible for antioxidant properties in fruit and vegetable juices (Han et al., 2007). Ademosun et al. (2020) studied the antioxidant properties of ginger‐based fruit drinks and concluded that juices containing higher concentrations of the ginger extract showed higher TP content. Also, ginger extract in combination with hibiscus solution in functional drinks was studied by Adesokan et al. (2013) and the results showed that they contain high concentrations of TP and antioxidants.

According to ANOVA, the *F*‐value of the model of 60.03 for DPPH scavenging property indicates that the antioxidant property of functional beverages was significantly dependent on the type and level of added extracts at *p* ≤ .05 and the quadratic regression equation for DPPH (Equation (7)) was significant.

Sample containing 1 ml HS, 0.5 ml CE, and 0.5 ml HS showed the highest antioxidant property (Equation (7)).

(7) $$\text{DPPH}=77.38A+86.27B+87.22C+33.09\text{AC}+31.64A^{2}\text{BC}‐956.56{\text{ABC}}^{2}$$

Where A = CE, B = GE, and C = HS.

The *R* ^2^ value = 0.9897 and adjusted *R* ^2^ value = 0.9732 indicated that this quadratic model had high efficiency.

The contour diagram for DPPH response has been presented in Figure 1b. The inhibition percent of DPPH of formulations ranged from 76.09% to 90.84% belonging to sample 5:0.83 ml CE, 0.83 ml GE, 0.33 ml HS and sample 14:0.5 ml CE, 1 ml GE, 0.5 ml HS, respectively. Ramadan‐Hassanien (2008) reported that the DPPH scavenging activity of popular commercial hibiscus drink in Egypt was 63.9% that is less than the minimum antioxidant activity of formulations in this study. It can be understood from the results that the radical scavenging activity of juices was more dependent on hibiscus concentration than ginger and cardamom amounts. It could be attributed to the high anthocyanin content of hibiscus extract. Comparison of antioxidant properties of sample 9 and sample 12 containing the same amount of hibiscus solution (sample 9:0.33 ml GE/0.83 ml CE/0.83 ml HS with inhibition percent of 85.11 ± 0.97 and sample 12:0.83 ml GE/0.33 ml CE/0.83 ml HS with inhibition percent of 83.19 ± 1.21%) showed that the second extract which is effective on antioxidant properties of the beverage is cardamom essential oil (Figure 1b) which could be attributed to high terpenic compounds of cardamon essential oil.

#### 3.4.2 Anthocyanin, Flavonoid, and vitamin C content of functional beverage formulations

**FIGURE 2:**
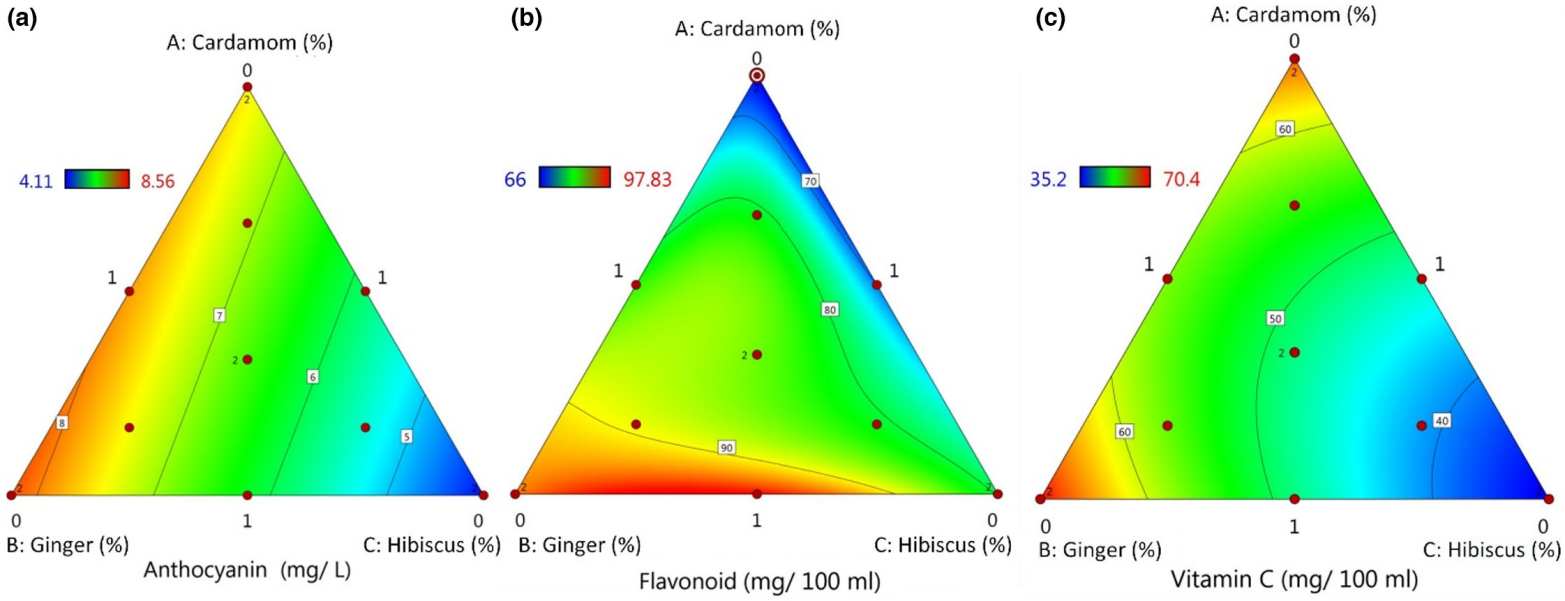
Plots showing the effect of ginger extract (GE), cardamom essential oil (CE), and hibiscus solution (HS) on anthocyanin content (a), flavonoid content (b), and vitamin C content (c) of functional beverages

Anthocyanin variations dependent on different additives were best described by the linear mixture model indicated in Equation (8). The *F*‐value of the model of 41.04 shows that the addition of different extracts into the juices was effective on the anthocyanin content. Also, the *p* ≤ .05 shows that the terms of the model are significant and represent the true system. Results showed that increasing the concentration of hibiscus and cardamom essential oil increases the anthocyanin content of functional beverages (Figure 2a). Anthocyanin content of different formulations of juices was within 4.11 and 8.56 mg/L.

(8) Anthocyanin=7.53A+8.22B+4.17C

Where A is CE, B is GE, and C is HS. *R* ^2^ = 0.8818, adjusted *R* ^2^ = 0.8603.

Escobar‐Ortiz et al. (2020) admitted that hibiscus solution includes a high amount of anthocyanins. Tashtoush et al. (2016) studied the temperature and solvent effect on the anthocyanin content of spices’ extracts. They reported that the anthocyanin content of green cardamom extract was 40.9 mg/100 g and of the ginger extract was 18.2 mg/100 g. Results of this study indicated that hibiscus and cardamom content was more effective than ginger extract on anthocyanin content of juices. Sample 9 containing 0.83 ml HS/0.83 CE/0.33 GE and sample 3 containing 1 ml CE/1 ml GE/0 HS showed the highest and less anthocyanin content, respectively.

The total flavonoid content of different formulations of juices ranged between 66.00 and 97.83 mg/100 ml (Figure 2b). ANOVA indicated that the *F*‐value of the model was 3164.89 and the *p* ≤ .05, which declared that the model is significant. The special quartic mixture model of total flavonoids could state 99.98% of the effect of different concentrations of additives on the total flavonoid content of the juice blends, as shown in Equation (9). The highest flavonoid content of 97.83 mg/100 g belonged to formulation 7:1 ml CE/0.5 ml GE/0.5 ml HS and the less flavonoid content of 66.00 mg/100 g was associated to formulation 4:0 CE/1 ml GE/1 ml HS.

(9) $$\text{Total flavonoid content}=65.98A+93.81B+79.64C+13.42\text{AB}‐11.56\text{AC}+44.10\text{BC}+716.88A^{2}\text{BC}‐387.24{\text{AB}}^{2}C‐296.88{\text{ABC}}^{2}$$

Where A= CE, B= GE, and C= HS; *R* ^2^ = 0.9998, adjusted *R* ^2^ = 0.9995. All interaction effects in the Equation (9) were significant at *p*‐value < .05. As can be understood from the results, the flavonoid content of juices was affected by CE concentration than other additives. Previous studies showed that cardamom extract and essential oil have a high content of flavonoids (Amma et al., 2010). On the other hand, the second content which was impressive on flavonoid content was HS. After CE, increase in HS concentration led to increase in the flavonoid content. Mak et al. (2013) reported that the hibiscus flowers encompass a high content of flavonoids and anthocyanin (2155.39 ± 112.6 mg/100 g of ethanolic extract). However, in this study, the concentration of GE was not as effective as for CE and HS, but previous investigations admitted the high content of flavonoids in GE (Ahmed Ali et al., 2018).

Vitamin C content of different fruit juice blends is best presented in Equation (10). This model could describe 97.86% vitamin C variation in juice formulations. The *F*‐value of the model was 73.20 and *p*‐value was less than .05, which indicated that the model is significant. The impact of different concentrations of additives in juice formulations on vitamin C content is presented by the contour plot (Figure 2c). Results have shown that the addition of HS increased the vitamin C content of juices. While the incorporation of CE and GE did not affect vitamin C content. Tyagi and Tyagi (2018) reported that vitamin C content of *Hibiscus rosa‐sinensis* flower extract is 26.98 mg/100 g. Therefore, high content of the vitamin C of HS can effectively increase the vitamin C content of functional beverages. As it is expected, vitamin C degrades at 70°C and this temperature is less than the one needed for the steam distillation process which is used for the extraction of essential oil and herbal extracts. So, it cannot be found in GE and CE. Therefore, the addition of GE and CE cannot increase the vitamin C content of juices. However, studies have shown that incorporating extract and essential oil can decrease vitamin C degradation rate in juices during storage time (Kapoor et al., 2011). Formulations containing 1 ml CE/0 GE/1 ml HS (sample 2) and 0 CE/1 ml GE/1 ml HS (sample 4) had high amounts of vitamin C (70.4 and 67.23 mg/100 ml) and sample 3:1 ml CE/1 ml GE/0 HS contain the least amount of vitamin C (35.2 mg/100 ml).

(10) Vitamin C content=67.11A+70.12B+34.72C‐46.06AB

Where A = CE, B = GE, C = HS. *R* ^2^ value = 0.9786, and adjusted *R* ^2^ = 0.9652. AC and BC interaction effect was not significant in Equation (10).

#### 3.4.3 Optimization and validation of optimal functional beverage

**FIGURE 3:**
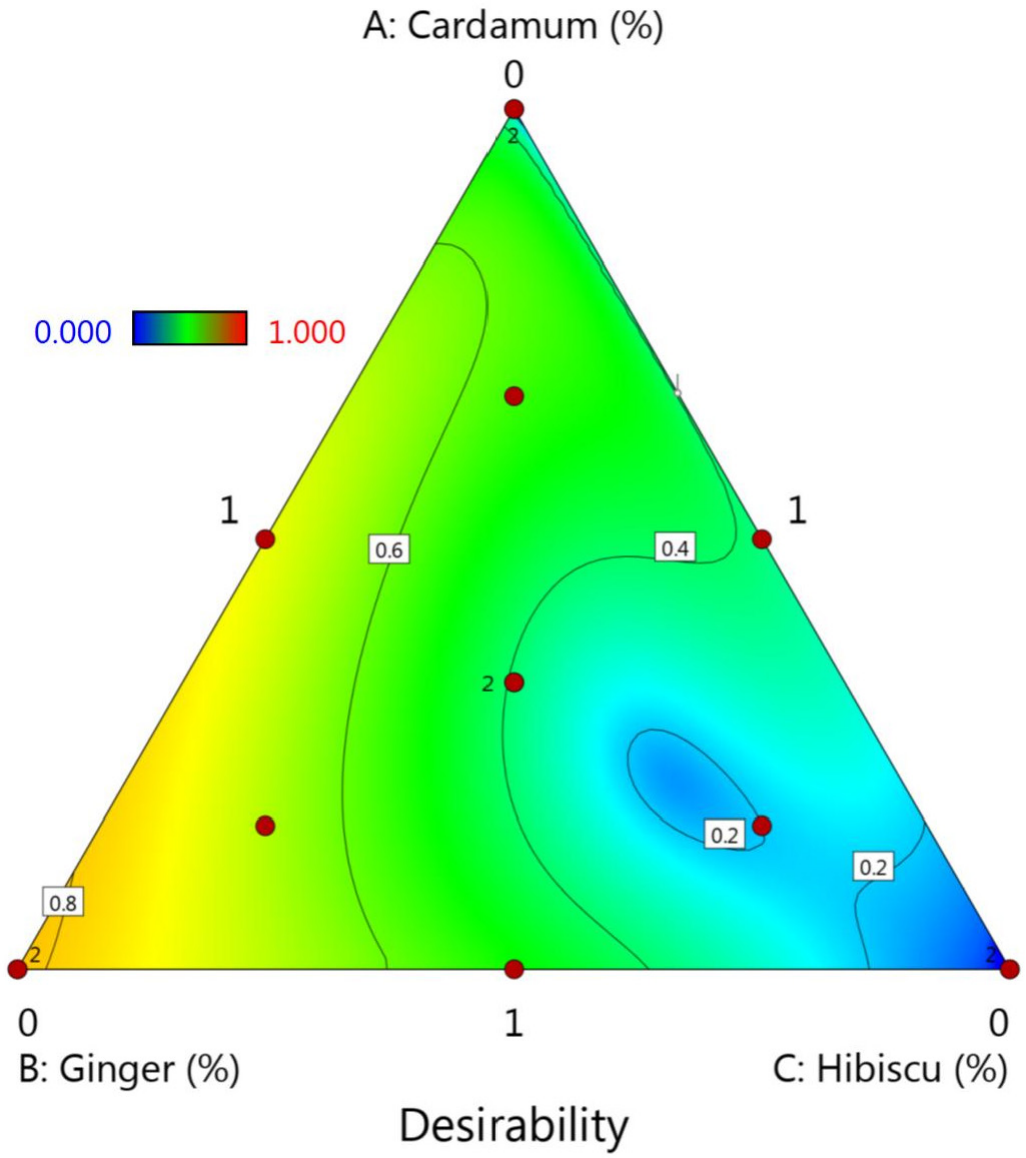
The plot of the desirability of responses for different functional beverage formulations

Results of mixture optimization analysis suggested three formulations to reach the maximum amounts of total phenol, DPPH scavenging ability, anthocyanin, flavonoid, and vitamin C content. Selected juice blend formulations were sample 2:1 ml CE/0 GE/1 ml HS (desirability of 0.81), sample 10:0.5 ml CE/0.5 ml GE/1 ml HS (desirability of 0.73), and sample 12:0.33 ml CA/0.83 ml GE/0.83 ml HS (desirability of 0.39), respectively (Figure 3). Since the plant extracts and essential oil used in this study have been selected for the production of nutraceutical drinks and the experiments performed in this study do not show all of their beneficial properties in preventing diseases, we chose the beverage that has all of the additives. Also, considering the desirability analyzed in the optimization process, the optimum formulation was selected as sample 10:0.5 ml CE/0.5 ml GE/1 ml HS. The mean experimental and predicted values of the selected optimized functional beverage were in good agreement with each other (Table 7).

**TABLE 7:** Predicted and experimental mean values of chemical properties of the optimal functional beverage

Sample 10:0.5 ml CE/0.5 ml GE/1 ml HS	Mean value	Total phenol (µg GAE/ml)	DPPH (%)	Anthocyanin (mg/L)	Flavonoid (mg/100 ml)	Vitamin C (mg/100 ml)
	Predicted mean	166.82	86.75	7.87	83.25	57.09
	Experimental mean	166.60 ± 1.57	87.02 ± 0.75	8.46 ± 0.53	83.33 ± 0.22	57.21 ± 2.30
	Percent error (%)	0.13	0.31	7.49	0.09	0.21

Abbreviations: CE, Cardamom essential oil; GE, Ginger extract; HS, Hibiscus solution.

## 4 CONCLUSION

According to the antioxidant properties, total phenol, anthocyanin, flavonoid, vitamin C content, and sensory analysis, the best fruit juice blend to produce a functional drink was 60% pomegranate juice/20% grape juice/20% barberry juice. It was decided that up to a maximum of 1 ml of GE (0.001 v/v) and 1 ml of CE (0.005 v/v) can be added to 99 ml of juices based on the overall acceptability of sensory properties, but the amount of addition of hibiscus had no limitation according to customer acceptability. According to the mixture design, the best model that could describe the total phenols, DPPH radical scavenging property, anthocyanin, flavonoid, and vitamin C content of juice formulations was the special quartic model. Whereas, linear and special quadratic models qualified better anthocyanin and vitamin C content variations, respectively. The optimum composition of juice formulation was selected based on each desired active component's content and antioxidant responses. Sample 10:0.5 ml CE/0.5 ml GE/1 ml HS was the optimum formulation. The TP content of the formulations was mainly influenced by GE content while CE content was effective on anthocyanin and flavonoid content. On the other hand, HS content was impressive on all of the functional properties of formulations. The optimum fruit juice formulation of this study can be used to produce a functional drink with high consumer acceptability and health benefits.

## CONFLICT OF INTEREST

We wish to confirm that there are no known conflicts of interest associated with this publication and there has been no significant financial support for this work that could have influenced its outcome.

## Data Availability

The data that support the findings of this study are available on request from the corresponding author. The data are not publicly available due to privacy or ethical restrictions.
